# Supplementary material for: The Moderating Role of Family Resilience on the Relationship between COVID-19-Related Psychological Distress and Mental Health among Caregivers of Individuals with Eating Disorders in Post-Pandemic China
Source: Int J Environ Res Public Health. 2023 Feb 15;20(4):3417. doi: 10.3390/ijerph20043417 (PMC9965091; doi:10.3390/ijerph20043417)
Supplement: Supplementary file 1 [file ijerph-20-03417-s001.zip › Supplementary File S1.pdf]

Supplementary File S1: Life disruptions experienced by caregivers as a result of COVID-19 (% of participants)

	Not at all	Somewhat/ Moderately	Quite a bit/ Extremely
1. To what extent has your family's life been disrupted by the COVID-19 pandemic?	11.9	61.7	26.4
2. Has your family experienced financial hardship?	46.8	45.8	7.5
3. Have you feared for your own or your family's health or safety?	9.0	58.7	32.4
4. Have your children feared for their own or their family's health or safety?	22.4	62.2	15.4
5. Has your family had difficulty getting food?	68.7	28.3	3.0
6. Has your family experienced a shortage of essential goods (e.g., toilet paper, cleaning supplies)	77.1	19.9	3.0
7. Has your family had difficulties finding childcare?	50.7	37.8	11.5
